# Neural Congruency Effects in the Multi-Source Interference Task Vanish in Healthy Youth after Controlling for Conditional Differences in Mean RT

**DOI:** 10.1371/journal.pone.0060710

**Published:** 2013-04-16

**Authors:** Kamin Kim, Joshua Carp, Kate D. Fitzgerald, Stephan F. Taylor, Daniel H. Weissman

**Affiliations:** 1 Department of Psychology, University of Michigan, Ann Arbor, Michigan, United States of America; 2 Department of Psychiatry, University of Michigan, Ann Arbor, Michigan, United States of America; Hangzhou Normal University, China

## Abstract

According to the conflict monitoring model of cognitive control, reaction time (RT) in distracter interference tasks (e.g., the Stroop task) is a more precise index of response conflict than stimulus congruency (incongruent vs. congruent). The model therefore predicts that RT should be a reliable predictor of activity in regions of the posterior medial frontal cortex (pMFC) that are posited to detect response conflict. In particular, pMFC activity should be (a) greater in slow-RT than in fast-RT trials within a given task condition (e.g., congruent) and (b) equivalent in RT-matched trials from different conditions (i.e., congruent and incongruent trials). Both of these effects have been observed in functional magnetic resonance imaging (MRI) studies of adults. However, neither effect was observed in a recent study of healthy youth, suggesting that (a) the model does not accurately describe the relationship between RT and pMFC activity in this population or (b) the recent study was characterized by high variability due to a relatively small sample size. To distinguish between these possibilities, we asked a relatively large group of healthy youth (n = 28) to perform a distracter interference task - the multi-source interference task (MSIT) - while we recorded their brain activity with functional MRI. In this relatively large sample, both of the model’s predictions were confirmed. We conclude that the model accurately describes the relationship between pMFC activity and RT in healthy youth, but that additional research is needed to determine whether processes unrelated to response conflict contribute to this relationship.

## Introduction

Response conflict is ubiquitous in everyday life. For example, when a policeman directing traffic indicates that a driver should go through a red light, the driver may experience conflict between the required response (pressing the gas pedal) and the automatic response (pressing the brake). Similarly, when a supervisor asks an employee whether she enjoyed a subpar company meal, the employee may experience conflict between the situation-appropriate response (politely saying “yes”) and the pre-potent response (truthfully saying “no”). As these examples illustrate, optimal performance in many everyday situations depends critically on mechanisms that detect and resolve response conflict.

According to the conflict monitoring model of cognitive control, the detection and resolution of response conflict rely on distinct brain regions. Specifically, the posterior medial frontal cortex (pMFC) signals the presence of response conflict to the dorsolateral prefrontal cortex (DLPFC) which, in turn, resolves conflict by increasing attention to task-relevant stimuli and responses [1], [2], [3]. Consistent with this model, pMFC activity is greater in incongruent than in congruent trials of the Stroop and flanker tasks [2], [4], [5], [6], [7], [8]. Further, the pMFC is functionally connected to the DLPFC more strongly in incongruent than in congruent trials [9], [10]. Finally, elevated pMFC activity is followed by faster next-trial reaction times (RTs) for incongruent trials, suggesting that heightened recruitment of processes that detect response conflict triggers greater cognitive control in the next trial [11].

A further claim of the model is that reaction time (RT) is a more precise index of response conflict than stimulus congruency [12]. Consistent with this claim, in adults, pMFC activity increases linearly with RT across trials within both the congruent and incongruent conditions [10], [13], [14]. Moreover, RT-matched congruent and incongruent trials evoke equivalent pMFC activity [13], [15]. An ongoing controversy in the literature concerns whether such effects uniquely index a process that detects response conflict or, instead, index one or more processes whose recruitment increases with time on task independent of response conflict (e.g., sustained attention, arousal, effort, etc.) [15]. While resolving this controversy should be a top priority of future studies, the goal of the present study was simply to establish whether such effects are also present in healthy youth.

Establishing whether such effects can be observed in healthy youth is important for two reasons. First, the pMFC is thought to undergo significant structural and functional maturation during the years of youth [16], [17]. Thus, it is possible that current models (e.g., the conflict monitoring model) do not account for pMFC activity in healthy youth as well as they account for such activity in healthy adults. Second, attention deficit hyperactivity disorder (ADHD), which often emerges during childhood or adolescence, is frequently associated with brain abnormalities involving the pMFC [18], [19], [20]. Thus, advancing our understanding of how pMFC activity varies with RT in healthy youth could eventually prove useful for distinguishing between healthy and abnormal development.

Recent findings from our laboratory suggest that pMFC activity may not increase with RT in healthy youth. Contrasting with our findings in adults, Carp et al. (2012) reported that pMFC activity in healthy youth did not increase linearly with RT within the congruent and incongruent conditions of the multi-source interference task (MSIT) [21]. Incongruent trials therefore produced greater activity than RT-equated congruent trials. Carp et al. (2012) suggested there might be an important developmental difference in the neural systems that detect response conflict. Another possibility, however, is that high variability in our relatively small sample (n = 18) did not allow us to observe a relationship between pMFC activity and RT. In line with this possibility, data from children are often more variable than data from adults [22], [23].

We therefore revisited the conflict monitoring model’s claim that RT better indexes response conflict than stimulus congruency using a larger sample of healthy youth (n = 28). We reasoned that if this claim does not hold in healthy youth, then, as in our previous study [21], we should fail to observe a positive relationship between pMFC activity and RT. Consequently, incongruent trials should still evoke greater activity than RT-equated (or RT-matched) congruent trials. On the other hand, if this claim does hold in healthy youth, then, with our larger sample, we should be more likely to observe a positive relationship between pMFC activity and RT. Consequently, incongruent trials might not evoke greater activity than RT-equated (or RT-matched) congruent trials.

## Methods

### Ethics Statement

All experimental procedures were approved by the University of Michigan’s Biomedical and Health Sciences Institutional Review Board and were in compliance with the Code of Ethics of the World Medical Association (Declaration of Helsinki). Finally, all procedures were fully described to the participants before they consented to take part in the study; written informed consent was obtained from the participants’ parents.

### Participants

Twenty-eight healthy youth (14 female; mean age, 13.6 yrs; age range, 8–18 years) participated in the experiment. As the data reported here were from a larger, independent study of pediatric patients and controls (which have not been published yet), each participant was evaluated with numerous scales, inventories, checklists, and questionnaires. All participants were healthy as confirmed during a structured clinical interview using the Kiddie-Schedule for Affective Disorders and Schizophrenia-Present and Lifetime Version (K-SADS-PL) [24]. Handedness was evaluated with the Edinburgh Handedness Inventory [25]. This inventory revealed that 21 participants were right-handed, one was left-handed, and six used both hands equally often. Other measures included the Obsessive–Compulsive Inventory-Revised (OCI-R), the Multidimensional Anxiety Scale for Children (MASC), the Child Depression Inventory (CDI), the Child Behavior Checklist (CBCL), the Social Communication Questionnaire (SCQ), and the Pubertal Developmental Scale (PDS). As these measures were not of primary interest, they will not be further discussed.

### Stimuli and Apparatus

Visual stimuli were generated using E-Prime (Psychology Software Tools, http://www.pstnet.com/eprime.cfm) and projected onto a translucent screen that participants viewed through a mirror attached to the head-coil. Participants responded by pressing one of three keys on an MR-compatible keypad.

### Task and Procedure

Participants performed an event-related version of the MSIT, which evokes robust conflict-related activity in the pMFC [13], [26], [27]. More specifically, in each trial, participants pressed one of three keys on an MR-compatible keypad to identify the unique digit (1, 2, or 3) in a string of three, horizontally-aligned alphanumeric characters. The digits 1, 2, and 3, respectively, were mapped to the index, middle, and ring fingers of the right hand. In congruent trials (i.e., 1××, ×2×, ××3), neither the unique digit’s spatial position nor the identity of the distracters conflicted with the correct response. In incongruent trials (e.g., 212, 322, 112), both the unique digit’s spatial position and the identity of the distracters conflicted with the correct response.

Participants performed five 3-minute runs of the MSIT. Each run contained 24 congruent trials, 24 incongruent trials, and 12 fixation trials presented in a random order. In each congruent and incongruent trial, the stimulus was presented for 500 ms, after which a fixation cross appeared for 2500 ms. In each fixation trial, a fixation cross was presented for 3000 ms. Prior to the scanning session, each participant completed a block of 48 practice trials outside the MR scanner. He or she completed an additional practice block if mean accuracy in the first practice block was lower than 70%. After each run of the scanning session, participants were instructed to respond more quickly in the next run if mean accuracy in the just-completed run exceeded 80%. Participants were instructed to respond more slowly and accurately in the next run if mean accuracy in the just-completed run did not exceed 50%. These instructions were designed to ensure adequate numbers of correct and error trials for subsequent data analyses (but only correct trials were analyzed in the present study). However, since nearly all of the participants achieved accuracy levels of 80% or higher in every run (with the exception of two participants whose accuracy fell between 70 and 80 percent in one run each), participants almost always received just one type of feedback (i.e., “respond more quickly in the next run”).

### MRI Data Acquisition

A 3T GE Signa MRI scanner was used to collect neuroimaging data. Functional images were collected using a reverse spiral sequence (repetition time (TR) = 2000 ms, echo time (TE) = 30 ms, flip angle (FA) = 90°; field of view = 20 cm; slices/volume = 40; slice thickness = 3 mm, voxel size = 3.44 × 3.44 × 3 mm). To allow for the equilibration of the blood oxygenated level-dependent (BOLD) signal in each run, the first four volumes of each run were discarded (no trials were presented in these volumes). Finally, high-resolution anatomical images (T_1_-overlay, three-dimensional spoiled gradient echo (SPGR); slice thickness = 1.5 mm, 0 mm skip) were collected to facilitate subsequent normalization of the functional images to Montreal Neurological Institute (MNI) space.

### Data Preprocessing

Physiological artifacts (i.e., respiration and heart beat) were removed from the time series of each run using the RETROICOR algorithm [28], [29]. Data preprocessing was performed using SPM8 (the Wellcome Department of Cognitive Neurology, London, UK, www.fil.ion.ucl.ac.uk) and consisted of several steps. First, the functional images were corrected for slice acquisition delays. Second, to correct for head motion, the functional images were spatially realigned to the first image of the first run. Third, each participant’s T_1_–overlay (anatomical) volume was co-registered to his or her functional images. Fourth, each participant’s high-resolution T_1_–SPGR was co-registered to his or her T_1_–overlay. Fifth, the T_1_–SPGR was normalized to the Montreal Neurological Institute (MNI) template (normalized voxel size, 3.75×3.75×3.0 mm). Sixth, the parameters generated to normalize the T_1_–SPGR were used to normalize the functional images. Seventh, the functional images were spatially smoothed with a 3-dimensional Gaussian filter (full-width at half maximum, 8 mm).

### Data Analysis

The preprocessed functional data were analyzed using the general linear model (GLM) as implemented by SPM8. In this model, the event-related BOLD responses evoked by correct congruent, correct incongruent, and error trials were modeled with separate regressors. A canonical hemodynamic response function was used to model the BOLD response in each trial. Response omissions and trials with RTs greater or less than three standard deviations from their conditional means (i.e., outliers) were modeled separately and excluded from subsequent analyses (1.80% of trials).

For each condition of interest (correct congruent, correct incongruent, and error trials), we included four parametric regressors. These regressors modeled the linear, quadratic, cubic, and quartic effects of reaction time (RT) on the BOLD signal. Each parametric RT regressor was generated using the mean-centered RTs from the corresponding condition of interest (e.g., correct congruent trials). Therefore, each RT regressor modeled trial-to-trial variations in the BOLD signal that were linked to trial-by-trial variations in RT in a single trial type. We included curvilinear terms in the model to verify that any observed relationships between RT and the BOLD signal (i.e., RT-BOLD relationships) were predominantly linear [10], [30], a critical assumption of the RT-regression analysis described below. To regress out BOLD signal changes that were correlated with head motion, we included twenty-four head-motion parameters as regressors of no interest (i.e., the linear, squared, derivative, and squared derivative of the six rigid-body movement parameters [31]). A high-pass filter (1/128 Hz) was applied to remove low-frequency noise from the time series data, and serial correlations were corrected using an autoregressive AR(1) model. Finally, random effects analyses were used to combine data across participants and to ensure that our findings would generalize to the population.

### Estimating and Controlling for the Effect of RT on Brain Activity

#### RT-regression analysis

We conducted an RT-regression analysis to (a) determine the relationship between RT and brain activity and (b) control for this relationship when contrasting activity in incongruent and congruent trials [13], [21]. To determine the relationship between RT and brain activity, we used the linear parametric RT regressors (see *Data Analysis*) to estimate the slope of the linear, trial-by-trial relationship between RT and the BOLD signal at each voxel (i.e., the RT-BOLD slope, 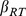), separately for each trial type. Positive values of the resulting beta coefficients would indicate greater activity in slow-RT than in fast-RT trials, consistent with the conflict monitoring model’s claim that slow-RT trials have greater response conflict than fast-RT trials [12].

To control for the relationship between RT and brain activity when contrasting activity in incongruent and congruent trials, we compared activity in incongruent trials to an estimate of activity in congruent trials with RTs equal to the mean RT in incongruent trials (i.e., CongruentEQ trials). To estimate activity in CongruentEQ trials, we first estimated the RT-BOLD slope (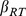) in congruent trials using RT-regression (see above). Next, we multiplied this slope by the difference in mean RT between incongruent and congruent trials. We then added the result to our regression-derived estimate of mean activity in congruent trials. Thus, the RT-equated congruent-trial BOLD signal was calculated as follows:
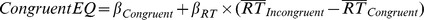



Finally, we compared activity in incongruent trials to activity in CongruentEQ trials. Equivalent activity in these conditions would fit with the conflict monitoring model’s assertion that RT-matched congruent and incongruent trials contain identical amounts of response conflict [12].

#### RT-matching analysis

As described above, the RT-regression analysis assumes that the RT-BOLD relationship is predominantly linear. We verified this assumption both in our prior work [10], [13], [30] and in the present study (see **RESULTS**). Nonetheless, we wished to determine whether RT-matched congruent and incongruent trials evoke equivalent pMFC activity with an approach that does not depend on this assumption. Therefore, we conducted a within-participant, RT-matching analysis.

The goal of the RT-matching analysis was to compare the BOLD signal in RT-matched congruent and incongruent trials (excluding errors and RT outliers). To define a subset of RT-matched trials in each participant, we implemented the following procedure. First, for each correct congruent trial, we identified all correct incongruent trials (across runs) with RTs that fell within 10 ms of the RT in the correct congruent trial. Second, assuming that one or more incongruent trials were identified, we chose just one trial to serve as the RT-matched partner of the correct congruent trial. This RT-matched incongruent trial was selected to maximize the total number of RT-matched pairs of congruent and incongruent trials.

After they were identified in each participant, the RT-matched trials were entered into a regression model. This model included separate regressors for (a) RT-matched congruent trials, (b) RT-matched incongruent trials, (c) non-matched congruent and incongruent trials, and (d) error trials. We reasoned that equivalent pMFC activity in RT-matched congruent and RT-matched incongruent trials would support the conflict monitoring model’s assertion that these trials contain equivalent response conflict.

#### RT-subsampling analysis

One drawback of the RT-matching approach above is that it reduces the total number of trials in the analysis. For this reason, failing to observe different levels of pMFC activity in RT-matched incongruent and congruent trials might reflect a lack of statistical power. To preclude this interpretation, we created a second subset of trials for each participant. Notably, this “RT-subsampled” data set preserved the behavioral congruency effect in the full data set but contained the same number of trials as the RT-matched data set. We reasoned that if neural congruency effects were present in the RT-subsampled data set, then the absence of such effects in the RT-matched data set could not simply reflect the reduced number of trials in the analysis.

For each participant, we selected the RT-subsampled trials as follows. First, we sorted correct trials by RT, separately for the congruent and incongruent conditions. Second, from each of these two RT-sorted lists of trials, we selected trials at uniform intervals in a manner that equated the number of selected trials to the number of RT-matched trials. For example, suppose that a hypothetical participant had (a) 110 correct congruent trials and 100 correct incongruent trials in the full data set and (b) 50 congruent and 50 incongruent trials in the RT-matched data set. In this example, our algorithm for including trials in the RT-subsampled data set would select approximately every second (i.e., 110/50^th^) correct congruent trial and exactly every second (i.e., 100/50^th^) correct incongruent trial, resulting in 50 pairs of RT-subsampled trials. After they were identified in each participant, the RT-subsampled trials were entered into a regression model. This model included separate regressors for (a) RT-subsampled congruent trials, (b) RT-subsampled incongruent trials, (c) all other correct trials, and (d) errors.

### Voxelwise Analysis Thresholds

Voxelwise maps were thresholded using the topological false discovery rate (FDR) correction for multiple comparisons (cluster-level corrected *p*<.005). We chose topological, rather than voxel-wise, FDR correction because it allows more appropriate interpretations of topological features (e.g., peaks and regions) of the activation signal [32], [33].

### Region of Interest (ROI) Analyses

The SPM toolbox Marsbar (http://marsbar.sourceforge.net/) was employed to conduct ROI analyses. First, we created an ROI that included all voxels within an 8 mm radius of coordinates of interest in the pMFC. Second, we averaged the fMRI signal across all voxels in the ROI, separately for each trial type. We considered *p*-values less than.05 (two-tailed) to be significant.

## Results

### Behavior

**Figure 1:**
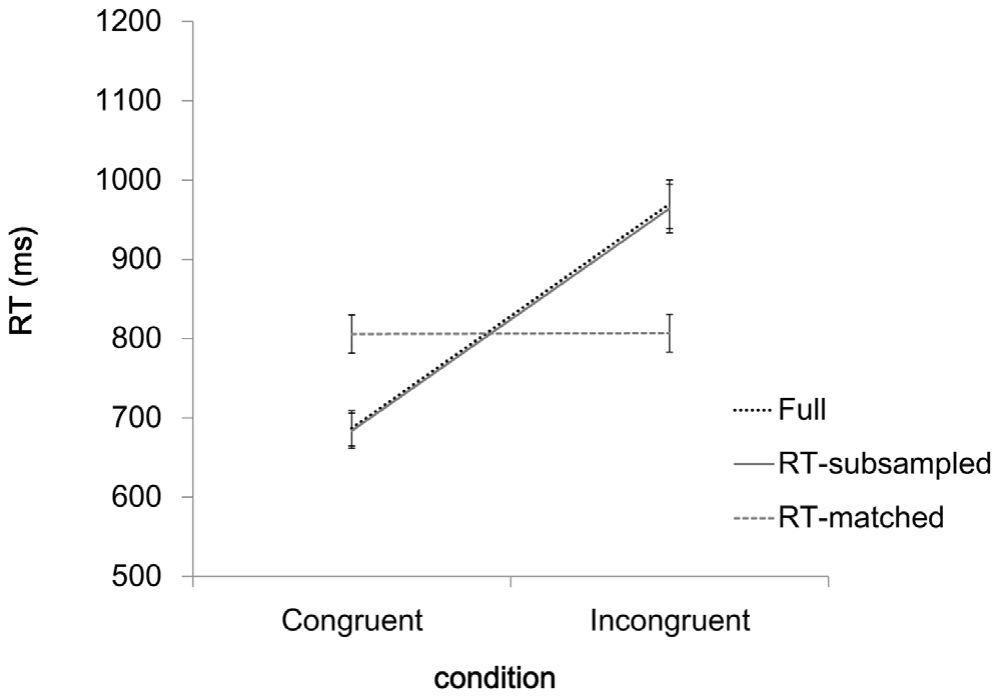
Behavioral congruency effects in the full, RT-matched, and RT-subsampled data sets. Error bars denote one standard error of the mean.

The behavioral data matched our expectations. First, replicating prior findings with the MSIT [26], [27], in the full data set, mean RT was longer in incongruent than in congruent trials (969.8 ms vs. 686.9 ms; *t*(27) = 18.211, *p*<.001; **Fig. 1**, Full). Analogously, mean error rate was higher in incongruent than in congruent trials (8.2% vs. 0.1%; *t* (27) = 7.218, *p*<.001). Second, as intended, the behavioral congruency effect was greatly reduced in the RT-matched data set (0.96 ms; *t*(27) = 3.77, *p* = .001; **Fig. 1**, RT-matched). Indeed, it was just 0.3% of the 282.9 ms congruency effect in the full data set. Third, and also as intended, the behavioral congruency effect in the RT-subsampled data set (280.5 ms; *t*(27) = 18.30, *p*<.001; **Fig. 1** **,** RT-subsampled) was similar in magnitude to the behavioral congruency effect in the full data set (282.9 ms). Table 1 presents the average number of trials that were included in each data set and the mean error rate for the full data set (the RT-matched and RT-subsampled data sets were not associated with error rates because they were composed entirely of correct trials). As expected, there were significantly more trials in the full data set (226) than in either the RT-matched data set (77, *t*(27) = 32.110, *p*<.001) or the RT-subsampled data set (77; *t*(27) = 32.110, *p*<.001).

**Table 1:** The average number of trials and average error rate (when applicable) for each analysis.

Analysis	Average number of trials	Average error rate
Full Data	226	4.12%
RT-Matched Data	77	N/A
RT-Subsampled Data	77	N/A

### fMRI

#### Effects of congruency and RT on brain activity

**Figure 2:**
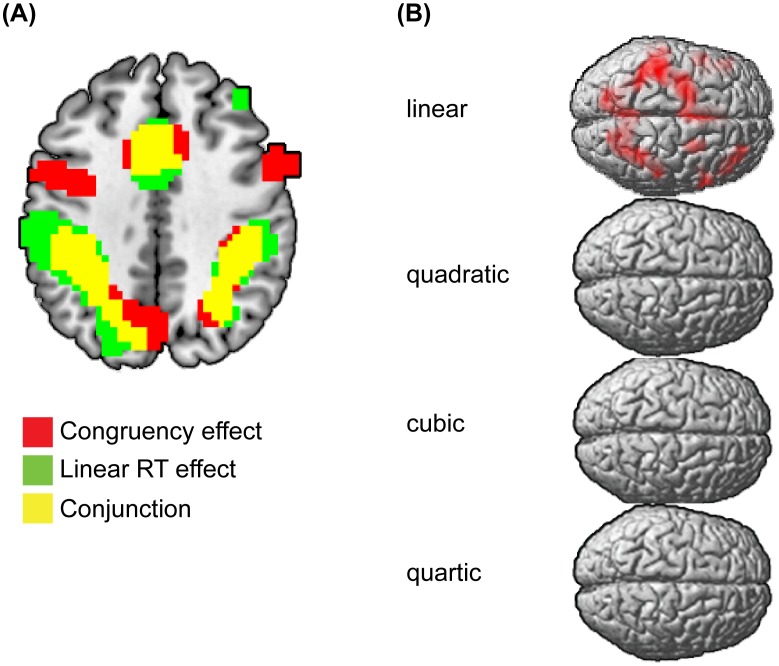
Effects of congruency and RT on brain activity. (A) Brain regions in which the BOLD signal was greater in incongruent than in congruent trials (red), increased linearly with RT (green), and brain regions in which both effects were observed (yellow). (B) A polynomial regression analysis of the effect of RT on the BOLD signal. The BOLD signal increased linearly with RT in numerous frontal and parietal regions. Whole-brain maps were thresholded using the topological false discovery rate (FDR) correction for multiple comparisons (cluster-level corrected *p*<.005).

Using the full data set, we conducted whole-brain analyses to identify regions activated by trial congruency (correct incongruent trials vs. correct congruent trials), regions in which activity varied linearly and positively with RT, and the conjunction of these two effects. Consistent with the conflict monitoring model of cognitive control [12] and replicating prior studies of adults [13], [15], both congruency and RT were associated with activity in the pMFC. First, activity was significantly greater in incongruent than in congruent trials in multiple frontal, parietal, and sensory regions including the pMFC (**Fig. 2A**, congruency effect; **Table 2**). Second, activity increased linearly with RT in numerous frontal and parietal regions including the pMFC (**Fig. 2A**, linear RT effect; **Fig. 2B**, linear; **Table 3**; nonlinear RT-BOLD relationships were not observed). Third, activity increased linearly with RT in many of the same brain regions that showed congruency effects including the pMFC (**Fig. 2A**, conjunction). Given this latter result, we next investigated whether the congruency effect in the pMFC could be explained by conditional differences in mean RT between incongruent and congruent trials. To do so, we used two complementary analyses: RT-regression and RT-matching.

**Table 2:** Brain regions in which activity was greater in incongruent than in congruent trials.

Region	∼BA	Number of voxels	MNI coordinates			FDR-corrected *p*-value		Peak *t*-value
			X	y	z	cluster-level	peak-level	
R. cingulate gyrus	32	728	4	15	40	.000	.005	8.09
R. medial frontal gyrus	32	728	4	8	52	.000	.006	7.58
L. medial frontal gyrus	6	728	−2	12	49	.000	.006	7.37
R. inferior frontal gyrus	–	90	49	5	25	.008	.011	6.89
L. inferior frontal gyrus	9	1477	−44	1	31	.000	.029	6.36
R. inferior parietal lobule	40	371	42	−33	43	.000	.043	5.94
R. precuneus	7	371	29	−57	52	.000	.043	5.81
L. precuneus	–	1477	−6	−74	43	.000	.006	7.47
R. middle occipital gyrus	19	308	46	−78	−11	.000	.097	5.16
L. inferior occipital gyrus	18	639	−47	−85	−5	.000	.043	5.93
R. cuneus	–	639	22	−98	−8	.000	.030	6.28
R. insula	13	175	42	12	1	.000	.142	4.86
L. culmen	–	49	−30	−50	−32	.049	.100	5.11

**Table 3:** Brain regions in which activity increased linearly with RT.

Region	∼BA	Number of voxels	MNI coordinates			FDR-corrected *p*-value		Peak *t*-value
			X	y	z	cluster-level	peak-level	
R. cingulate gyrus	32	2548	4	8	46	.000	.110	5.49
L. cingulate gyrus	32	2548	−6	15	34	.000	.127	5.32
L. medial frontal gyrus	–	2548	−2	1	52	.000	.048	6.12
R. middle frontal gyrus	–	76	25	8	61	.013	.347	4.19
L. middle frontal gyrus	6	2548	−26	−9	64	.000	.014	7.08
L. inferior frontal gyrus	–	345	−54	8	28	.000	.127	5.34
R. inferior frontal gyrus	–	252	60	15	28	.000	.252	6.83
L. precuneus	–	2548	−9	−74	49	.000	.052	6.00
L. postcentral gyrus	40	2548	−40	−30	46	.000	.014	6.93
L. inferior parietal lobule	–	2548	−37	−40	43	.000	.010	7.69

#### RT-regression analysis

Consistent with the conflict monitoring model, a whole-brain analysis did not reveal greater pMFC activity in incongruent trials than in RT-equated, CongruentEQ trials. In fact, this analysis revealed no significant activations in the entire brain volume.

However, as shown in Figure 2A (congruency effect), some voxels exhibited a significant congruency effect but not a significant RT-BOLD relationship. These voxels were located in various sub-regions of the midline frontal cortex (including regions of the pMFC), the medial and superior frontal gyri, the precuneus, and the occipital lobe. Given that these voxels exhibited a significant congruency effect in the absence of a significant RT-BOLD relationship, it is possible that small congruency effects remained in these voxels after RT correction but did not survive the stringent corrections for multiple comparisons that we applied in the whole-brain analysis. To investigate this possibility, we conducted an additional voxelwise analysis, which was restricted to these voxels. Although this analysis was biased to reveal congruency effects that survived RT correction, it did not reveal greater activity in incongruent than in CongruentEQ trials in any brain regions.

**Figure 3:**
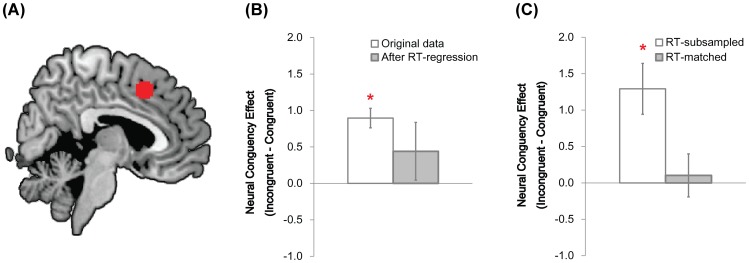
ROI analyses in the pMFC. (A) The pMFC ROI (x = 2, y = 16, z = 46) overlaid on the Ch2bet template in MNI space. (B) RT-regression analysis. In the full data set, there was a significant congruency effect in the pMFC ROI as indexed by significantly greater activity in incongruent than in congruent trials (light grey bar). However, this congruency effect vanished after controlling for conditional differences in mean RT. Specifically, mean activity in incongruent trials did not differ from a regression-derived estimate of activity in RT-equated congruent trials (dark grey bar). (C) RT-matching and RT-subsampled analyses. Analogous to the RT-regression analysis, pMFC activity did not differ between RT-matched incongruent and congruent trials (dark grey bar). However, pMFC activity was greater in RT-subsampled incongruent trials than in RT-subsampled congruent trials (light grey bar).

To further increase statistical power for observing small congruency effects in the RT-corrected data, we conducted ROI analyses in the pMFC (see **METHODS**). The first analysis involved an independently-defined pMFC ROI (x = 2, y = 16, z = 46; **Fig. 3A**) that a prior meta-analysis implicated in interference processing [34]. As shown in Figure 3B, one-sample t-tests revealed a significant congruency effect in the original data (*t*(27) = 6.66, *p*<.001) that was no longer present in the RT-equated data (*t*(27) = 1.11, *p*>.2). Thus, as in the voxelwise analyses above, incongruent trials did not evoke significantly greater activity than CongruentEQ trials. Additional analyses revealed there were no higher-order relationships between RT and activity in this ROI (i.e., quadratic, cubic, or quartic; all *p*s >.05).

Considering the wide age range of the participants (i.e., 8–18 years), it is possible that the congruency effect in this ROI varied with age. To investigate this possibility, we conducted two univariate analyses of variance (ANOVAs) with congruency serving as the dependent variable and mean-centered age serving as a covariate. These ANOVAs replicated the t-tests above: there was a significant congruency effect in the original data (*p*<.001) but not in the RT-equated data (*p*>.2). Additional analyses revealed no significant effect of age on the congruency effect (all *p*s >.1). Thus, independent of age, incongruent trials did not evoke significantly greater activity than CongruentEQ trials.

Finally, it is possible that small congruency effects remained in the RT-corrected data but only in pMFC regions that showed maximal congruency effects in the original data. To investigate this possibility, we created three additional pMFC ROIs, each of which was centered on a peak of maximal activation in the original voxelwise contrast of incongruent versus congruent trials (see the first three rows of **Table 2** for the exact coordinates of these ROIs). Although subsequent ROI analyses were biased to reveal congruency effects that survived RT correction, incongruent trials did not evoke significantly greater activity than CongruentEQ trials in any of these ROIs (all *p*s >.1). Thus, even in pMFC regions that showed maximal congruency effects in the original data, incongruent trials did not evoke greater activity than CongruentEQ trials.

#### RT-matching analysis

**Figure 4:**
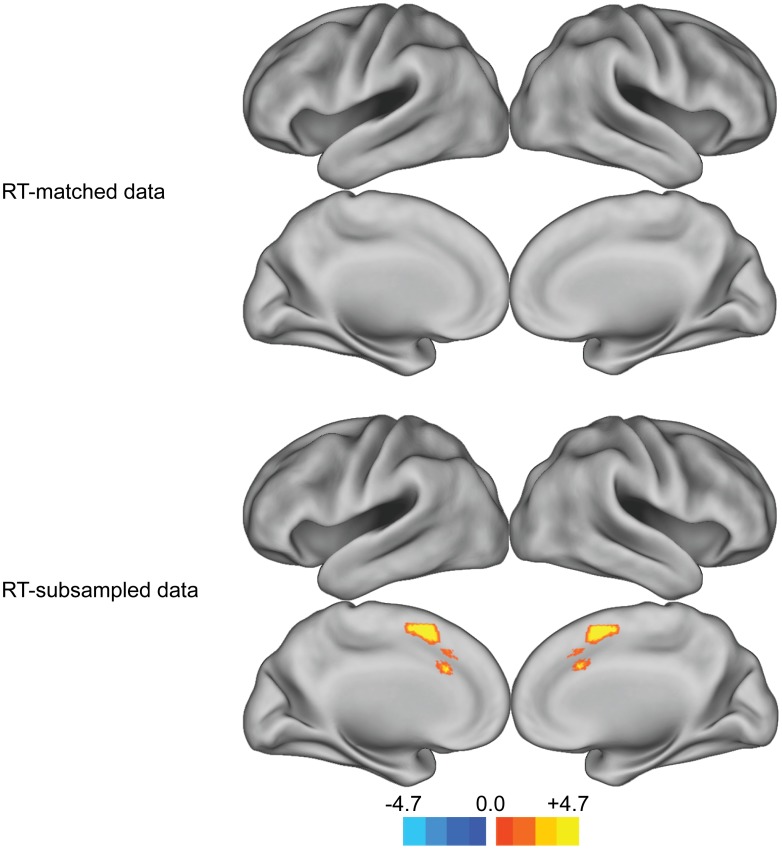
Whole-brain analyses of congruency effects in the RT-matched and RT-subsampled data sets. A significant congruency effect was observed in the RT-subsampled data set, but not in the RT-matched set. Whole-brain maps were thresholded using the topological false discovery rate (FDR) correction for multiple comparisons (cluster-level corrected *p*<.005).

We first investigated whether incongruent and congruent trials that were naturally matched for RT evoked different activity in the pMFC. Consistent with the RT-regression analysis, a whole-brain analysis revealed no difference in mean pMFC activity between RT-matched incongruent and congruent trials (**Fig. 4**, RT-matched data).

We next investigated whether RT-matched incongruent trials evoked greater pMFC activity than RT-matched congruent trials in a subset of voxels that showed a significant congruency effect but not a significant RT-BOLD relationship (**Fig. 2A**, congruency effect). Although this voxelwise analysis was biased to yield congruency effects that survived RT-correction, it did not reveal greater activity in RT-matched incongruent trials than in RT-matched congruent trials.

Finally, to further increase statistical power for identifying small congruency effects that may have survived RT-correction, we conducted ROI analyses in the pMFC. First, we probed the independently-defined pMFC ROI that was identified in a prior meta-analysis of interference processing [34] (x = 2, y = 16, z = 46; **Fig. 3A**). Consistent with the voxelwise analyses above, there was no significant difference in mean activity between RT-matched incongruent and congruent trials (*t*(27) = 0.35, *p*>.1). Second, we probed the three pMFC ROIs that showed maximal congruency effects in the original data (for exact coordinates, see the first three rows of **Table 2**). Although this analysis was biased to reveal congruency effects that survived RT-correction, mean activity in RT-matched incongruent and congruent trials did not differ significantly in any of these ROIs (all *p*s >.5).

#### RT-subsampling analysis

The RT-matched data set contained far fewer trials than the full data set: on average, the RT-matching process removed 66% of the correct trials in each participant (see **Table 1**). For this reason, the absence of congruency effects in the RT-matched data set may have resulted from a reduction of statistical power due to the reduced number of trials in the RT-matched data set, relative to the full data set. To investigate this possibility, we created an RT-subsampled data set that contained the same number of trials as the RT-matched data set but preserved the behavioral congruency effect in the full data set (see **METHODS** and *Behavior*).

A whole-brain analysis comparing mean activity in RT-subsampled incongruent and congruent trials revealed congruency effects in the pMFC (**Fig. 4**, RT-subsampled data). Thus, in these regions of the pMFC, the absence of a congruency effect in the RT-matched data set was more likely due to the fact that congruent and incongruent trials were matched for RT than to the fact that fewer trials were included in the RT-matched data set than in the full data set.

ROI analyses involving the RT-subsampled data set further supported this interpretation. First, we probed the independently-defined pMFC ROI that was identified in a prior meta-analysis of interference processing [34] (x = 2, y = 16, z = 46; **Fig. 3A**). Unlike in the RT-matching analysis, we observed a significant congruency effect in this ROI (*t*(27) = 3.70, *p*<.005). Second, we probed the three pMFC ROIs that showed maximal congruency effects in the original data, just as we did in the RT-regression and the RT-matching analyses. Unlike in those prior analyses, we observed a significant congruency effect in each of these ROIs (all three *p*s <.005). Third, univariate ANOVAs revealed a significant congruency effect in each of the four ROIs above even when age was included as a covariate (all *p*s <.005). They also revealed no effect of age on the magnitude of the congruency effect (all *p*s >.1). These results further suggest that the absence of significant congruency effects in the RT-matched data set was due to the fact that incongruent and congruent trials were matched for RT rather than to the fact that the RT-matched data set contained fewer trials than the full data set.

Finally, we sought converging evidence that the neural congruency effect in the pMFC is driven by conditional differences in mean RT. To this end, we conducted a ROI analysis to determine whether the congruency effect was significantly smaller in the RT-matched data set than in the RT-subsampled data set in the independently-defined ROI we probed earlier (x = 2, y = 16, z = 46; **Fig. 3A**). More specifically, we conducted an ANOVA with congruency (congruent, incongruent) and sampling method (RT-matched, RT-subsampled) as within-participants factors. As expected, we observed a significant main effect of congruency (*F*(1, 27) = 8.99, *p*<.01). Most important, we observed a significant interaction between congruency and sampling method: replicating our prior work in adults [13], the congruency effect was smaller in the RT-matched data set than in the RT-subsampled data set (*F*(1, 27) = 6.96, *p*<.02; **Fig. 3C**). This finding provides converging support for the view that neural congruency effects in the pMFC stem, at least partly, from conditional differences in mean RT.

## Discussion

### The Relationship between pMFC Activity and RT in Healthy Youth

The conflict monitoring model posits that RT is a direct index of response conflict [12]. In line with this view, two prior studies of adults have reported that activity in brain regions thought to detect response conflict is (a) greater in slow-RT trials than in fast-RT trials within a given trial type (e.g., congruent) and (b) equivalent in RT-matched congruent and incongruent trials [13], [15]. In the present study, we revisited the question of whether these effects are absent in healthy youth [21] while employing a larger sample size to better ensure that the absence of such effects would not be due to high variability in this population [22], [23]. Contrary to our prior findings, both effects were observed, thereby confirming that the conflict monitoring model accurately describes the relationship between pMFC activity and RT in healthy youth.

We used two complementary approaches to determine whether interference-related pMFC activity (i.e., correct incongruent>correct congruent) was eliminated by controlling for conditional differences in mean RT. First, we used an RT-regression analysis to compare activity in incongruent trials to an estimate of activity in congruent trials with RTs equal to the mean RT in incongruent trials. Second, we used an RT-matching approach to contrast activity in incongruent and congruent trials that were naturally matched on RT. In both cases, controlling for conditional differences in mean RT eliminated interference-related activity in the pMFC. Further revealing that conditional differences in mean RT are essential for observing neural congruency effects, interference-related activity in the pMFC was significantly greater when incongruent and congruent trials were not matched for RT than when they were matched. These findings replicate previous studies of adults [13], [15]. They also fit with the conflict monitoring model’s assertion that RT is a better index of response conflict than stimulus congruency.

Our findings therefore establish that conflict-related activity in the pMFC is driven by RT in healthy youth, similar to what is observed in healthy adults [13], [15]. From a developmental perspective, this outcome suggests that qualitatively similar RT-related mechanisms drive conflict-related pMFC activity in these two age groups. Of importance, this outcome could not be predicted *a priori* because the pMFC is thought to undergo significant structural and functional maturation during the years of youth [16], [17]. Also important, this outcome may have implications for our understanding of clinical disorders in youthful populations that are characterized by abnormal pMFC activity (e.g., ADHD [18], [19], [20]). In particular, it suggests the possibility that abnormally large or small RT-BOLD relationships in the pMFC might ultimately help to distinguish youth with these disorders from healthy controls.

### The Present Findings in Relation to a Prior Study of Conflict Processing in Healthy Youth

Our findings are partly inconsistent with the results of a prior study published by our group [21]. In our prior study, pMFC activity in healthy youth did not increase with RT in the same task employed here (our earlier study also documented differences in the magnitude of the RT-BOLD relationship between healthy youth and adults, but the present study only considered healthy youth and so did not evaluate this finding). Thus, in an RT-regression analysis, incongruent trials evoked greater pMFC activity than RT-equated congruent trials (i.e., CongruentEQ trials).

We can only speculate as to why a significant RT-BOLD relationship in the pMFC was absent in our previous study of healthy youth but present in the current study. First, there could have been high variability due to the relatively small sample size (n = 18); notably, the present study employed a considerably larger sample (n = 28). Second, the relationship between RT and pMFC activity may vary with overall levels of task performance. In line with this possibility, mean RT, mean error rate, and the mean difference in RT between incongruent and congruent trials were numerically higher in our prior study than in the present one. However, these between-study differences did not achieve conventional levels of significance (all *p*s >.1). Third, the participants in the present study may have been older than those in our prior study [21], leading them to exhibit a more “adult-like” (i.e., larger) RT-BOLD relationship in the pMFC. Weighing against this possibility, however, the average age of participants in the present study was slightly younger than that in Carp et al. (2012) (13.6 years vs. 14.0 years). In sum, the source of the discrepancy between our prior findings and the present results is not easy to identify. Thus, additional studies will be needed to determine the factors that influence the presence of the RT-BOLD relationship in healthy youth.

### Caveats and Limitations

An important limitation of the present study is that one of its main findings confirms, rather than rejects, a null hypothesis. Specifically, our finding of equivalent pMFC activation in RT-matched, or RT-equated, incongruent and congruent trials confirms the null hypothesis of no activation differences between these conditions. This leaves open the possibility that small congruency effects were present even after controlling for conditional differences in mean RT and that such effects could be revealed by increasing statistical power (e.g., by increasing the number of participants). While we cannot rule out this possibility, we also found that conditional differences in pMFC activity between incongruent and congruent trials were significantly smaller in the RT-matched data set than in the RT-subsampled data set. Thus, conditional differences in mean RT at least partly explain conditional differences in pMFC activity.

Another important limitation of the present study is its interpretation of RT-BOLD relationships. While the present findings are consistent with the conflict monitoring model, pMFC activity could increase with RT for any of multiple reasons. These include (but are not limited to) increased demands on processes underlying sustained attention [35], [36], [37], autonomic arousal [38], and cognitive effort [39]. Thus, additional research is needed to distinguish conflict and non-conflict interpretations of the RT-BOLD relationship in the pMFC. Similarly, additional research is needed to understand why activity in frontal and parietal regions increases with RT more generally. RT-BOLD relationships in frontal and parietal regions have been repeatedly documented in fMRI studies [10], [14], [40] and occur in overlapping brain regions across a wide variety of tasks [37]. These relationships led Yarkoni and colleagues (2009) to suggest that “if two experimental conditions differ substantially in mean RT, a corresponding difference in frontal activation is likely to be observed *irrespective* of any other differences in task structure [37]”. In other words, when two conditions differ on mean RT, a corresponding difference in brain activity could reflect either (a) the cognitive process that is thought to distinguish the two conditions (e.g., response conflict) or (b) any other process whose recruitment varies with RT (e.g., sustained attention). From this perspective, conditional differences in mean RT are a serious and widespread confound in the cognitive neuroscience literature. Future work identifying the processes that underlie RT-BOLD relationships may therefore be crucial for advancing our knowledge of how different frontal and parietal regions contribute to cognition.

### Conclusion

In sum, the present findings reveal that pMFC activity in healthy youth varies with RT as predicted by the conflict monitoring model of cognitive control. They also suggest that a relatively large sample is needed to observe relationships between RT and BOLD signal in the developing pMFC, likely because data from healthy youth are more variable than data from adults. Finally, they indicate the need for additional studies to distinguish among numerous possible interpretations of the RT-BOLD relationships that we and others have observed.
